# From low to high latitudes: changes in fatty acid desaturation in mammalian fat tissue suggest a thermoregulatory role

**DOI:** 10.1186/s12862-019-1473-5

**Published:** 2019-07-26

**Authors:** Alicia I. Guerrero, Tracey L. Rogers

**Affiliations:** 1 Evolution and Ecology Research Centre, School of Biological, Earth and Environmental Sciences, University of New South Wales, Sydney, 2052 Australia; 2 Centro de Investigación y Gestión de Recursos Naturales (CIGREN), Instituto de Biología, Facultad de Ciencias, Universidad de Valparaiso, Gran Bretaña, 1111, Valparaiso, Chile

**Keywords:** Adipose tissue, Blubber, Environment, Fur, Insulation, Latitude, Acclimatization, Macroecology

## Abstract

**Background:**

Most fatty acids (FAs) making up the adipose tissue in mammals have a dietary origin and suffer little modification when they are stored. However, we propose that some of those FAs, specifically those that can be synthesised or modified by mammals, are also being influenced by thermal forces and used as part of the mechanism to regulate core body temperature. As FA desaturation increases, adipose tissues can reach colder temperatures without solidifying. The ability to cool the superficial fat tissues helps create a thermal gradient, which contributes to body heat loss reduction. Therefore, it is expected that animals exposed to colder environments will possess adipose tissues with higher proportions of desaturated FAs. Here, through a model selection approach that accounts for phylogeny, we investigate how the variation in FA desaturation in 54 mammalian species relates to the thermal proxies: latitude, physical environment (terrestrial, semi-aquatic and fully-aquatic) and hair density.

**Results:**

The interaction between the environment (terrestrial, semi- or fully-aquatic) and the latitude in which the animals lived explained best the variation of FA desaturation in mammals. Aquatic mammals had higher FA desaturation compared to terrestrial mammals. Semi-aquatic mammals had significantly higher levels of desaturated FAs when living in higher latitudes whereas terrestrial and fully-aquatic mammals did not. To account for dietary influence, a double bond index was calculated including all FAs, and revealed no correlation with latitude in any of the groups.

**Conclusions:**

We propose that FA modification is an important component of the thermoregulatory strategy, particularly in semi-aquatic mammals. Potentially this is because, like terrestrial mammals, they experience the greatest air temperature variations across latitudes, but they lack a thick fur coat and rely primarily on their blubber. Unlike fully-aquatic mammals, extremely thick blubber is not ideal for semi-aquatic mammals, as this is detrimental to their manoeuvrability on land. Therefore, the adipose tissue in semi-aquatic mammals plays a more important role in keeping warm, and the modification of FAs becomes crucial to withstand cold temperatures and maintain a pliable blubber.

**Electronic supplementary material:**

The online version of this article (10.1186/s12862-019-1473-5) contains supplementary material, which is available to authorized users.

## Background

In spite of the thermal challenges of the aquatic medium, mammals have colonised this environment on seven separate occasions and have sucessfully adapted [1]. Mammals exploit a wide range of thermal habitats, from the tropics to the poles, and from high altitudes to deep oceans; thus, they possess varied mechanisms to regulate their core body temperature. Changes in body size [2], hair density [3], metabolic rate [4], blubber thickness [5, 6], and blubber thermal conductivity [7] are some of these mechanisms, which have been well studied. However, the importance of the biochemical composition of the adipose tissue to mammalian thermoregulatory strategy has received less attention and yet is likely to be important, particularly where mammals use blubber as a thermal barrier.

Unlike other tetrapods, mammals have different insulation strategies, from fully furred through to totally naked forms, each with different thermal benefits and constraints. We propose that where mammals do not use fur, the adipose tissue is expected to play a more important role in keeping warm. In bare-skinned mammals, the cooling of the body surface in contact with air or water is of critical importance in order to reduce the heat lost to the environment. In cold superficial tissues occurs peripheral vasoconstriction, where the flow of warm blood is reduced [8], thus reducing body heat loss and creating a thermal gradient through the adipose tissue.

But cooling the body surface to temperatures similar to those of the ambient could create a potential problem. In extremely cold environments, low temperatures could cause rigidity and solidification of superficial tissues; potentially it is here when the biochemical structure of fats becomes more important.

Adipose tissues contain fat stored mostly in the form of triacylglicerol, which contains three molecules of fatty acids (FAs) [9]. The degree of desaturation of a FA is given by its number of double bonds within the chain [10]. As the degree of desaturation increases the tissues become more fluid and can reach colder temperatures for longer periods without solidifying [11]. An adipose tissue layer with higher degree of FA desaturation (FAs containing double bonds) is, therefore, better suited for lower temperatures as it is more effective for the maintenance of a thermal gradient.

The greatest contributor to adipose tissue FA composition is direct deposition of FAs from diet [9]. However, the FA composition of consumers rarely matches exactly that of their diet [12]; which suggests that there are other factors influencing FA metabolism. Since mammals have the ability to synthesize some of their FAs de novo or modify them [10], they do not necessarily have a direct dietary origin. Thus, it is possible to distinguish between dietary and endogenous FAs [13]. Dietary FAs are those that could only be derived from the diet as animals are unable to synthesize them, such as most polyunsaturated FAs (PUFAs) [14, 15]. Endogenous FAs are those that can be readily synthesized by the animal; most of them are saturated (SFAs) and monounsaturated FAs (MUFAs), although some of them may be partially obtained from diet [13, 14].

Although the properties of the adipose tissue will largely depend on the type of diet consumed, there is some evidence that supports the idea of a thermal influence in the metabolism of FAs. For example, northern aquatic and terrestrial mammals have higher proportions of MUFAs in their cold extremities compared to the rest of the body [15], suggesting that FA desaturation takes place when tissues are maintained at low temperatures to avoid solidification. Similarly, thirteen-lined ground squirrels exhibit higher MUFA-to-SFA ratios in their adipose tissues in winter compared to summer [16]. This suggests that certain changes in the FAs making up the adipose tissue are not only driven by diet but also by thermal acclimatization [15, 17–19]. The aim of this study was to evaluate whether thermal forces can have an effect on endogenous FAs in mammals. We hypothesise that animals living in colder environments, or those animals not relying on a fur coat, will have higher levels of FA desaturation in their adipose tissues.

To assess the hypothesis of thermal influence in FAs, we investigated the differences in degree of FA desaturation across a total of 54 mammalian species from terrestrial, semi-aquatic and fully-aquatic environments. We also investigated the patterns of FA desaturation across mammals inhabiting different latitudinal regions. The direct effect of ambient temperature is difficult to assess, since there is variation with season, water depth, and additionally, semi-aquatic mammals use two environments where depending on their lifestyle they can spend substantially more time in one than another. Therefore, in this study latitude was used as a coarse proxy for ambient temperature.

Fur-covered animals have skin temperatures closer to their body core whereas hairless animals have skin temperatures closer to those of the surrounding environment [20]. As most semi-aquatic mammals rely on a combination of both fur and blubber [19]; we investigated whether their FA desaturation is related to hair density.

We report here the patterns of FA desaturation as a function of latitude, in mammals inhabiting different physical environments, and discuss the potential drivers.

## Methods

### Fatty acid data

In total, we included *n* = 54 mammalian species in this analysis. A database of FA composition for mammalian species (*n* = 49 species) was collated from the literature, sourced from the online databases ScienceDirect and Google Scholar, using the search terms ‘fatty acids’, ‘lipids’, ‘adipose tissue’, ‘fat’ and ‘blubber’. Where available, only the FA values of the outer blubber layer were used, as FAs within this layer are less influenced by diet [12, 18, 21]. Where data was obtained from juveniles and adults, only adults data was used. When FA values of females and males were reported separately, only values from males were used, as females may experience changes in FAs associated with pregnancy and lactation (Additional file 1: Table S1.). For terrestrial mammals, only white adipose tissue data were used.

The FA composition from other 5 species (3 otariids: subantarctic fur seal, *Arctocephalus tropicalis*; New Zealand fur seal, *Arctocephalus forsteri*; and Australian sea lion, *Neophoca cinerea*; and 2 cetaceans: pygmy right whale, *Caperea marginata*; and Risso’s dolphin, *Grampus griseus*) was analysed from 0.3 g samples of outer blubber, using the SM Budge, SJ Iverson and HN Koopman [9] method to extract total lipids and prepare FA methyl esters. Gas chromatography analysis was performed as described in AI Guerrero and TL Rogers [22]. Blubber samples were sourced from animals stranded along the coast of Sydney, Australia. The FA composition of these species is available in Additional file 2: Table S2.

### Fatty acid desaturation

To determine the degree of desaturation of endogenous FAs we calculated a desaturation index (∆9-DI). This indicates to what extent potentially endogenous MUFAs could have been synthesized by modification of their corresponding SFAs [19]. PUFAs were not included in this analysis, as most have a dietary origin [15]; therefore differences in proportions of PUFAs are likely to be a result of differences in diet. Using the percentage by weight per FA, a ∆9-DI was calculated according to the formula of A Käkëla and H Hyvärinen [15]:

$$ \mathbf{\Delta }\mathbf{9}-\mathbf{DI}\kern0.5em =\frac{\left(\mathbf{wt}\%\mathbf{14}:\mathbf{1}\boldsymbol{\upomega } \mathbf{5}+\mathbf{wt}\%\mathbf{16}:\mathbf{1}\boldsymbol{\upomega } \mathbf{7}+\mathbf{wt}\%\mathbf{16}:\mathbf{1}\boldsymbol{\upomega } \mathbf{9}+\mathbf{wt}\%\mathbf{18}:\mathbf{1}\boldsymbol{\upomega } \mathbf{9}+\mathbf{wt}\%\mathbf{18}:\mathbf{1}\boldsymbol{\upomega } \mathbf{7}\right)}{\left(\mathbf{wt}\%\mathbf{14}:\mathbf{0}+\mathbf{wt}\%\mathbf{16}:\mathbf{0}+\mathbf{wt}\%\mathbf{18}:\mathbf{0}\right)} $$

where wt%, is the percentage by weight of the respective FA.

To account for the effect of dietary FAs on the desaturation of fat tissues, we calculated a double bond index (DBI), which includes all MUFAs and PUFAs, using the following formula [23, 24]:

$$ DBI=\frac{\sum x\left( wt\%\right)}{100} $$

where *wt%*, is the percentage by weight of each FA and *x* is its number of double bonds.

### Explanatory variables

The traits environment and latitude were collected for all 54 species in the database. Environment was categorised as terrestrial, semi-aquatic or fully-aquatic; where terrestrial mammals (*n* = 15) are those whose complete life cycle takes place on land only; semi-aquatic species (*n* = 25) are those who rely on both land (or ice) and water for breeding and feeding; and fully-aquatic species (*n* = 14) are those who spend their entire lives in water. Latitude corresponds to the location where the blubber samples were collected.

Hair density values, number of hairs per area (mm^2^), were obtained from the literature for most semi-aquatic mammals (*n* = 22), using the search terms ‘fur density’ and ‘hair density’ plus the species name, on ScienceDirect and Google Scholar. When primary and secondary hair densities were calculated separately, we added them up and used total hair density. Values were log-transformed for analysis.

### Data analysis

We used a model selection approach to test whether factors could explain the variation of ∆9-DIs (potentially endogenous FAs) or DBI (total FAs) in mammals. Initially, with the entire dataset (*n* = 54 species), we included the factors environment, latitude, or a combination of these variables, and then, with a smaller dataset of 22 semi-aquatic mammals we included the factors latitude, fur density, or a combination of these variables.

In detail, for the entire mammalian FA desaturation dataset (∆9-DI or DBI) we tested the following models: (a) an additive model containing the two variables (β_0_ + β_environment_ + β_latitude_); (b) an environment and latitude interaction model (β_0_ + β_environment_*β_latitude_); models where each variable would be the sole predictor for the desaturation of FAs: (c) a latitude model (β_0_ + β_latitude_) or (d) environment model (β_0_ + β_environment_); and (e) a Null model (β_0_).

Another series of models tested the relationship between hair density, latitude and ∆9-DI in semi-aquatic mammals. For this data subset, we tested: (a) an additive model containing two variables (β_0_ + β_latitude_ + β_hair_density_); (b) models to test for different slopes of hair density with latitude (β_0_ + β_latitude_*β_hair_density_); and (c) a hair density model (β_0_ + β_hair_density_), (d) a latitude model (β_0_ + β_latitude_) and (e) a Null model (β_0_).

To identify the model(s) with best support, we calculated the Akaike’s information criterion (with a correction for sample size; *AICc*) and the Akaike weight [25–27] for each model.

To account for the potential that phylogenetic relatedness confounds the variation in mammalian ∆9-DIs or DBI, we used phylogenetic generalised least squares (PGLS) analysis [28]. PGLS uses a lambda (λ) value, an estimate of phylogenetic correlation, that varies between 0 (indicating phylogenetic independence) and 1 (indicating that variation of traits between species covary proportionally with their phylogenetic relatedness) [29, 30]. We used the S Faurby and J-C Svenning [31] mammalian supertree and included 1000 random iterations to resolve possible polytomies. Two trees were pruned to include only the species present in our dataset, the first including all mammals for which we had data (*n* = 54), the second tree including only the semi-aquatic mammals for which hair density data was available (*n* = 22). The R (version 3.0.1) [32] packages, ‘phytools’ [33] and ‘caper’ [34] were used for tree manipulation and PGLS analyses, respectively. For models with the highest support, we extracted the 95% confidence intervals (CIs) and significance was deemed when the CIs did not overlap 0.

In order to identify which individual FAs drive changes in desaturation, when ∆9-DI was found to be significantly correlated with latitude, we applied linear regression analyses to those individual SFAs and MUFAs used to calculate the ∆9-DI.

Stratification of blubber FAs has been reported for most marine mammals [22] where the outer layer usually has greater proportions of MUFAs and smaller amounts of SFAs compared to the inner layer. Thus, to determine whether the use of different blubber sections (whole blubber core vs outer layer) would affect our analyses, we run an independent PGLS analysis for pinniped species for which either the outer layer (*n* = 14) or the whole blubber (*n* = 6) was used. We did not do the same for fully-aquatic mammals, as most FA values were obtained from the outer layer, with only two exceptions (Additional file 2: Table S2.).

Values are presented as mean ± standard deviation (SD) unless otherwise noted.

## Results

### Desaturation of endogenous fatty acids

The desaturation of endogenous FAs, ∆9-DI, was best explained by the interactive model (Table 1) including environment and latitude (β_0_ + β_environment_*β_latitude_). This model received the most support and accounted for 33% of the variance (*r* = 0.57), although the additive model including environment and latitude (β_0_ + β_environment_ + β_latitude_) had ∆AICc values within 2 units, which suggests that this model was equally supported. The Akaike weights showed that the interactive model was 2.7 times more likely to be the driver of mammalian ∆9-DI than the additive model. The PGLS λ value was 0.47; suggesting that the variation of ∆9-DI is not entirely dependent of phylogeny.

**Table 1:** A comparison of the level of support for possible explanatory models that describe the variation in desaturation of endogenous (Desaturation index: ∆9-DI) and total fatty acids (Double bond index: DBI) in mammals (*n* = 54)

Model	∆ AICc	PGLS λ	Effect size (r)	Weighted AICc
Desaturation index (∆9-DI)				
β_0_+ β_latitude_ * β_environment_	0.0	0.47	0.57	0.65
β_0_+ β_latitude_ + β_environment_	2.0	0.44	0.50	0.24
β_0_+ β_latitude_	4.2	0.61	0.36	0.09
β_0_+ β_environment_	7.0	0.37	0.38	0.02
β_0_	9.1	0.56	–	0.01
Double bond index (DBI)				
β_0_+ β_environment_	0.0	0.00	0.70	0.61
β_0_+ β_latitude_ + β_environment_	1.2	0.00	0.69	0.33
β_0_+ β_latitude_ * β_environment_	4.6	0.00	0.72	0.06
β_0_	14.0	0.68	–	0.00
β_0_ + β_latitude_	16.3	0.68	0.04	0.00

The results are produced from phylogenetic generalised least squares (PGLS) analysis across 1000 alternate resolutions of mammalian phylogeny

**Fig. 1:**
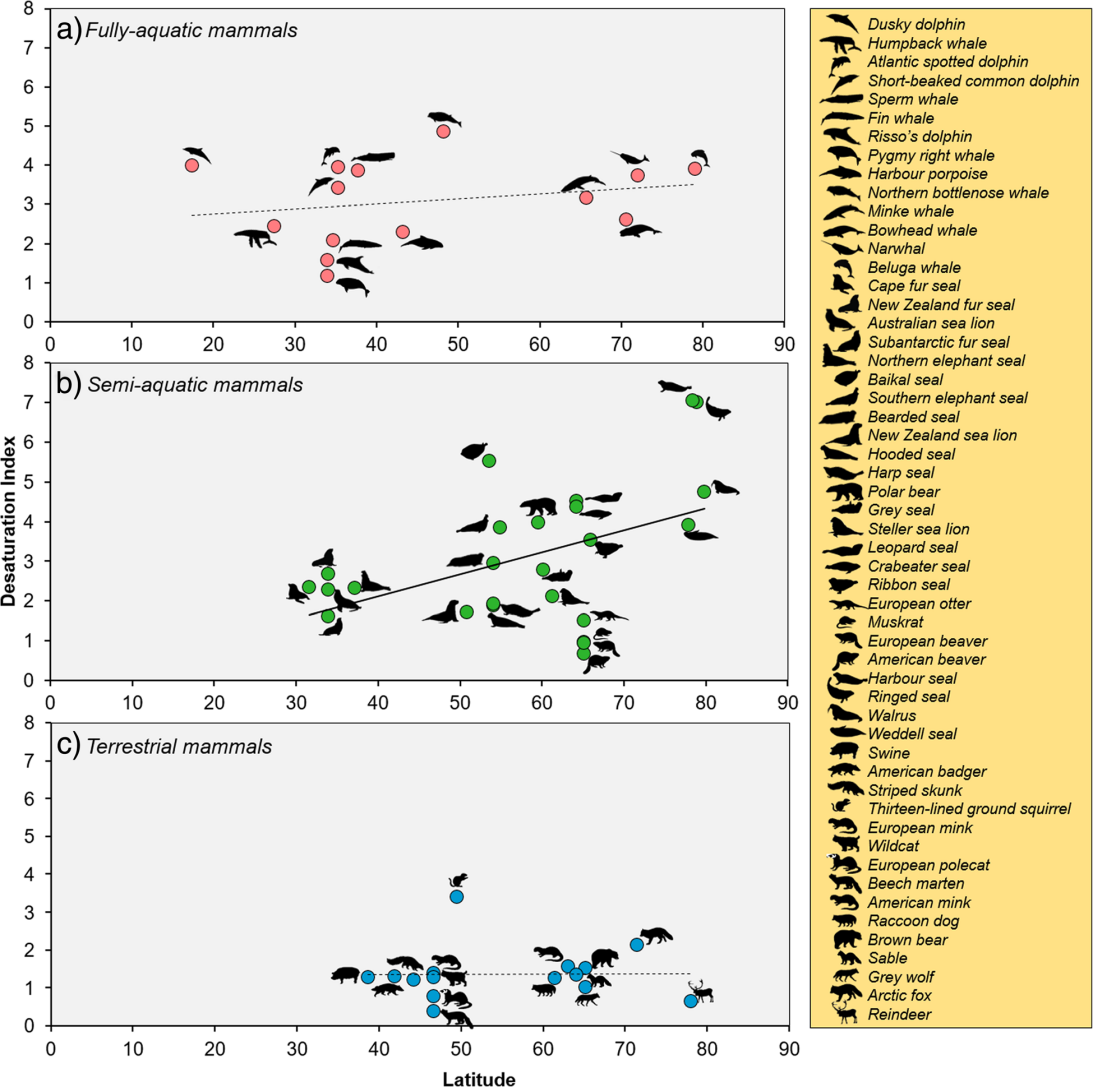
Desaturation index of fatty acids as a function of latitude for mammals inhabiting different environments. Dashed (non-significant correlation) and solid (significant correlation) lines indicate the phylogenetic generalised least squares (PGLS) regression lines for: **a** fully-aquatic (*y* = 0.02(*x*) + 2.27; *n* = 14); **b** semi-aquatic (*y* = 0.06(*x*) − 1.34; *n* = 25); and **c** terrestrial mammals (*y* =  − 0.01(*x*) + 1.62; *n* = 15). Only the blubber of semi-aquatic mammals displayed significantly (CIs = 0.02, 0.11) higher fatty acid desaturation when living in colder latitudes. Silhouettes are listed in the same order as they appear in each plot, from left to right and top to bottom. Scientific names can be found in Additional file 1: Table S1

Fully-aquatic mammals have a mean ∆9-DI of 3.08 ± 1.07, whereas semi-aquatic mammals have a ∆9-DI of 3.09 ± 1.75, and terrestrial mammals have a lower ∆9-DI of 1.37 ± 0.70. Among the three environments, only the ∆9-DI of semi-aquatic species had a significant positive correlation (CIs do not overlap 0) with latitude (slope = 0.06, CIs = 0.02, 0.11), such that semi-aquatic mammals at higher latitudes have significantly greater Δ9-DI values than species at lower latitudes (Fig. 1b). In comparison, the Δ9-DI values of fully-aquatic (slope = 0.02, CIs = − 0.02, 0.05) and terrestrial (slope = − 0.01, CIs = − 0.08, 0.05) mammals do not show a relationship with latitude (Fig. 1a and c).

**Fig. 2:**
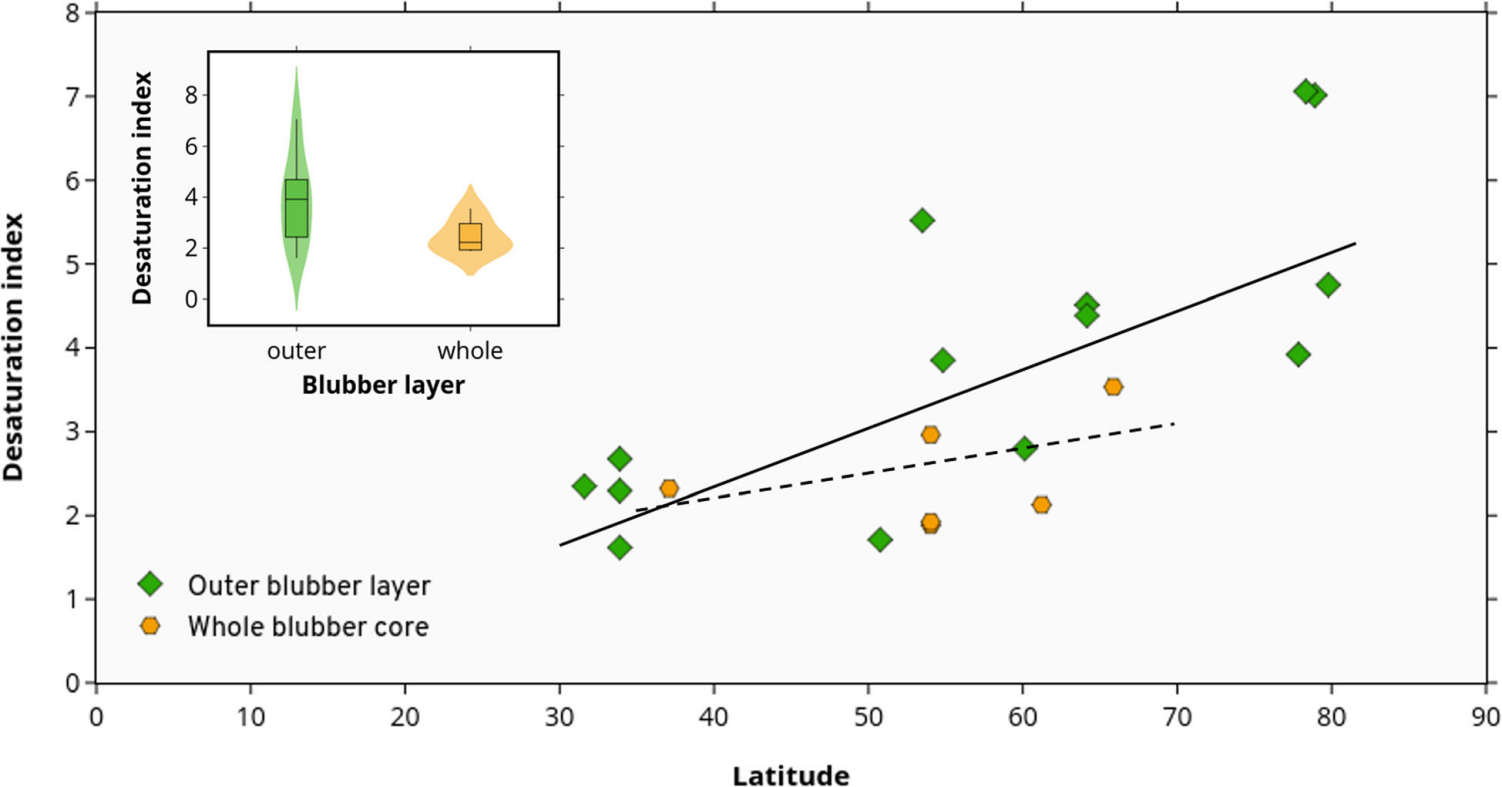
Desaturation index of blubber fatty acids as a function of latitude for pinnipeds, separated by blubber section used for analyses: whole blubber core (from skin to muscle; *n* = 6) or only the outer layer (section just beneath the skin; *n* = 14). Based on phylogenetic generalised least squares (PGLS), the dashed regression line indicates a non-significant correlation for whole blubber fatty acids (*y* = 0.03(*x*) + 1.01) whereas the solid line indicates significant correlation for outer blubber fatty acids (*y* = 0.07(*x*) − 0.41)

Pinnipeds for which only the outer layer was used, still displayed a significant correlation between Δ9-DI values and latitude (slope = 0.07, CIs = 0.04, 0.11; Fig. 2); however, those pinniped species for which the whole blubber core was used, displayed a non-significant but positive correlation between Δ9-DI and latitude (slope = 0.03, CIs = − 0.03, 0.09).

### Desaturation of total fatty acids

The DBI including all dietary and endogenous desaturated FAs was best explained by the environment model (β_0_ + β_environment_), which received the most support, explaining 50% of the variance (*r* = 0.70). The other model with ∆AICc value within 2 units was the additive model (β_0_ + β_environment_ + β_latitude_), which suggests that this model is equally supported; however, the addition of latitude only explains an additional variance of 0.1%. In addition, environment model was 1.8 times more likely to be the driver of DBI in mammals than the additive model (Table 1). The PGLS λ value was closer to 1 (0.70), suggesting that DBI covaries in proportion to the degree of shared evolutionary history.

**Fig. 3:**
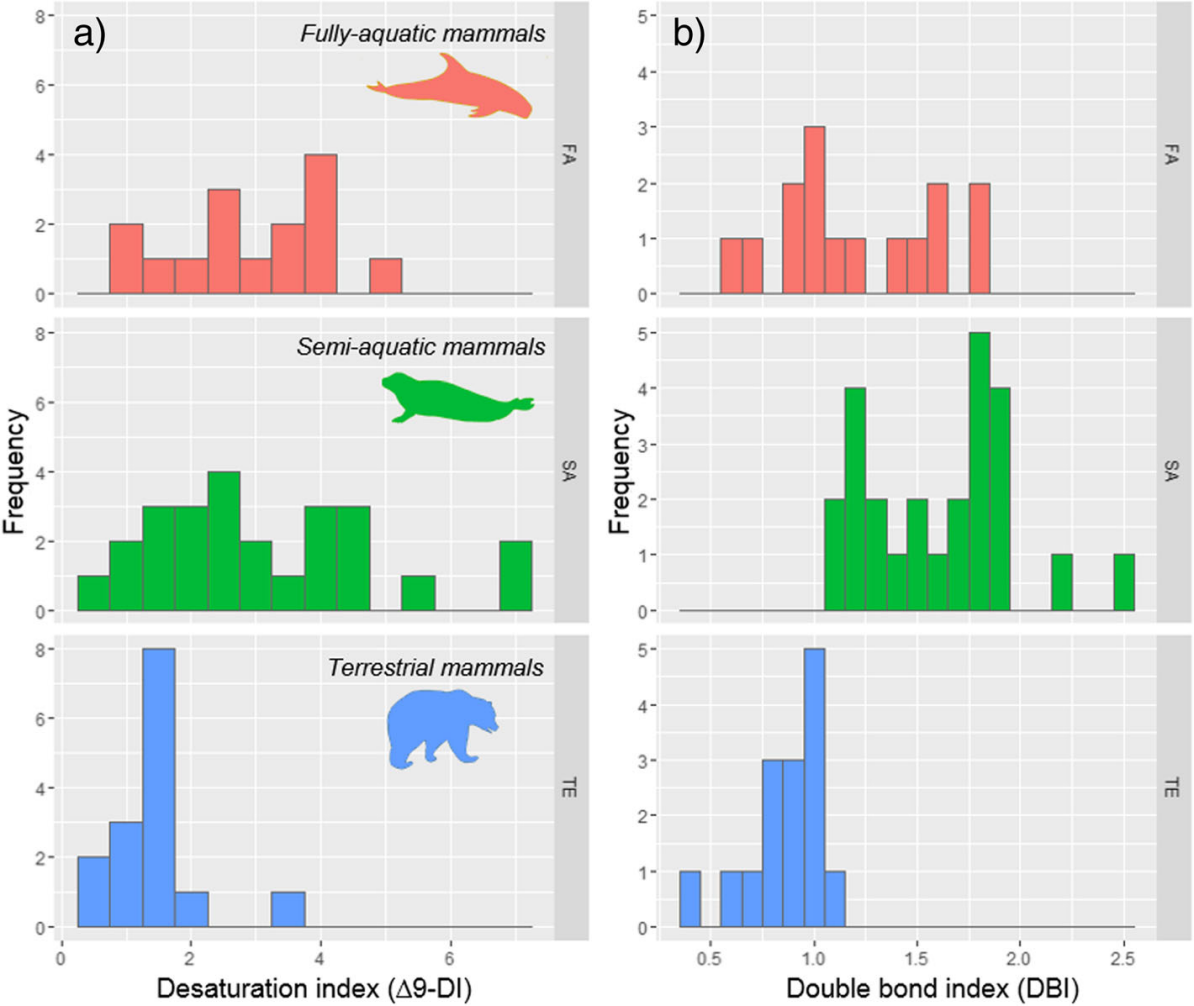
Histogram of **a** desaturation (∆9-DI) and **b** double bond index (DBI) of terrestrial (TE), semi-aquatic (SA), and fully-aquatic (FA) mammals. DBI has been calculated using all monounsaturated and polyunsaturated fatty acids, whereas ∆9-DI was calculated using only endogenous fatty acids

Fully-aquatic mammals have a mean DBI of 1.25 ± 0.36, whereas semi-aquatic mammals have a DBI of 1.61 ± 0.36 and terrestrial mammals a lower value of 0.85 ± 0.18 (Fig. 3). Intercept values were significantly different between fully-aquatic and terrestrial mammals (CIs = − 0.67, − 0.18), and between fully-aquatic and semi-aquatic mammals (CIs = 0.11, 0.55).

### Effect of hair density and latitude on fatty acids of semi-aquatic mammals

When we included hair density as a variable for the ∆9-DI of semi-aquatic mammals, we found that the model with the best support was the latitude model (β_0_ + β_latitude_, Table 2), which explains 35% of the variance (*r* = 0.60). The PGLS λ was 0.45; and there were no other models within 2 ∆AICc units. Including these 22 species, there was a positive significant correlation between latitude and ∆9-DI (slope = 0.08, CIs = 0.03, 0.12). Hair density only explained an additional 2% of the variance in the second-best model (β_0_ + β_hair_density_ + β_latitude_), which had an ∆AICc of 2.5.

**Table 2:** A comparison of the level of support for possible explanatory models that describe the variation in desaturation of endogenous fatty acids (Desaturation index: ∆9-DI) in semi-aquatic mammals (*n* = 22)

Model	∆ AICc	PGLS λ	Effect size (r)	Weighted AICc
β_0_+ β_latitude_	0.0	0.45	0.60	0.67
β_0_+ β_latitude_ + β_hair_density_	2.5	0.53	0.61	0.19
β_0_+ β_latitude_* β_hair_density_	4.1	0.00	0.63	0.09
β_hair_density_	6.0	0.00	0.38	0.03
β_0_	6.5	0.22	–	0.03

The results are produced from phylogenetic generalised least squares (PGLS) analysis across 1000 alternate resolutions of mammalian phylogeny

### Variation of individual fatty acids with latitude

**Fig. 4:**
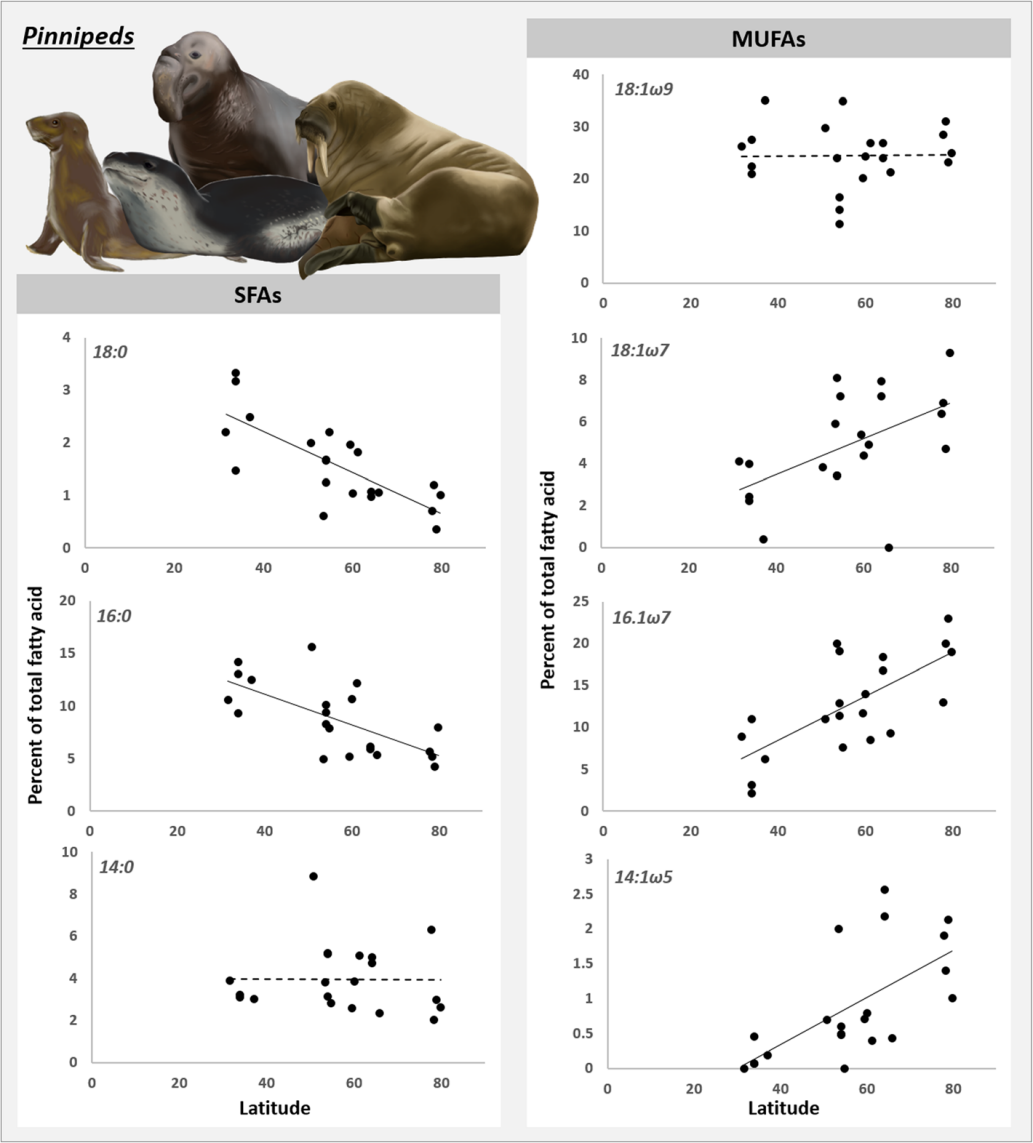
Percentage of saturated (SFAs) and monounsaturated (MUFAs) fatty acids as a function of latitude, for pinniped species. Dashed trend lines indicate no significant correlation between latitude and percent of fatty acid, and solid lines indicate significant correlation. These fatty acids were used to calculate the desaturation index

Linear regression analysis was only applied to pinniped species, as the correlation between ∆9-DI and latitude seems to be a pinniped-only pattern. Most MUFAs increased significantly (*P* < 0.01) with latitude whereas most SFAs (*P* < 0.01) decreased significantly (Fig. 4). Only the FAs 14:0 (*P* = 0.98) and 18:1ω9 (*P* = 0.93) did not show variation with latitude.

## Discussion

We show that the environment is potentially an important driver of FA desaturation in mammals. The variable environment was consistently important whether dietary FAs were considered (DBI), or not (∆9-DI). Conversely, latitude was an important driver only for ∆9-DI. The latitude in which the mammal lived was important for semi-aquatic mammals but it was not for terrestrial and fully-aquatic mammals. This study provides insights to whether this is only because the environment offers different FAs through its food webs, or also due to the demands each environment imposes which leads to intrinsic modification of FAs.

### Effects of latitude and environment on fatty acids

The best model of ∆9-DI included an interaction between environment and latitude. Aquatic mammals had higher degrees of desaturation than terrestrial mammals (Fig. 1), as expected. The aquatic medium has higher cooling potential than air [35], which suggests that the biochemical composition of adipose tissues plays a more important role in thermoregulation in aquatic mammals. Additionally, aquatic mammals use blubber as primary insulator whereas most terrestrial species use fur.

In fully-aquatic mammals, although there was a small positive correlation between ∆9-DI and latitude, this was not significant (Fig. 1a). Because fully-aquatic mammals rely solely on their blubber as a thermal insulator [19], this outcome was not expected. In bare-skinned animals, the skin and superficial tissues become very cold when they are exposed to low environmental temperatures [4]. In whales, for example, the skin temperature has been found to equal that of the water [20]. Therefore, the question is, why do fully-aquatic mammals not display greater degree of FA desaturation when they live in higher (colder) latitudes?

There are limitations to the study that could help us understand the absence of a pattern. Although the relationship between desaturation and latitude was positive, it was not significant, which can be due to the small sample size. In addition, we used whole blubber FAs for two of the species living in the highest latitudes: the narwhal, *Monodon monoceros*, and the beluga whale, *Delphinapterus leucas* (Fig. 1); whereas for all other fully-aquatic species we used the outer blubber layer. Since the blubber of marine mammals is stratified, where MUFAs are more abundant in the outer layer and SFAs in the inner layer [22], the use of whole blubber FAs should reduce the MUFA-to-SFA ratio leading to a decrease in ∆9-DI. If we had the data of the outer layer ∆9-DI instead, the values should be higher which could have led to a significant correlation. Unfortunately, it is difficult to measure the magnitude of the effect of the blubber section utilised, due to the small sample size.

Nevertheless, there is an aspect of FA composition not accounted for in this study. Certain families of cetaceans synthesise considerable levels of the endogenous isovaleric acid, a short branched-chain FA [36]. This FA has an extremely low melting point of − 37.6 ºC [37], and it has been found in great quantities (e.g.: ~ 35% in Hector’s dolphin, *Cephalorhynchus hectori*) in the outer blubber layer of some odontocetes [36]. They are hypothesised to play a role in maintaining blubber pliability in cold environments as they have been found to be more abundant in species inhabiting colder waters [9, 36]. Therefore, potentially some fully-aquatic mammals would rather synthesise this or other branched-chain FAs than increase their desaturation levels. Still, the synthesis of this FA is not ubiquitous among cetaceans; therefore, this does not completely explain the little variation of desaturation with latitude.

However; do fully-aquatic mammals need to maintain a pliable blubber in cold environments? The need for a pliable blubber might not be such for cetaceans. Some toothed whales (e.g. beaked and sperm whales) have FAs in the form of wax esters instead of triacylglycerols [38]; these wax esters have relatively high melting points [39], which makes them solid at most of the temperatures they find in the surrounding water. This implies that these species have rather rigid blubber layers, which does not seem to be an inconvenient for them.

Terrestrial mammals displayed low values of ∆9-DI and DBI, compared to the other groups (Fig. 3) and they did not exhibit a relationship between ∆9-DI and latitude (Fig. 1c). Although terrestrial mammals living in arctic climates encounter great diurnal and seasonal temperature variations [40], they seem to have little need for lowering the solidifying point of their adipose tissue. Our observations concur with those of PF Scholander, V Walters, R Hock and L Irving [41], who state that body fat does not seem to play any role in the insulation of terrestrial arctic mammals. In fact, fur has been found to be a more efficient insulator than adipose tissue [41], since it creates the same thermal gradient between body core and body surface, but across a much smaller thickness [19].

In the case of animals with poor insulating fur (thin, sparse fur or hairless), the thermal role of adipose tissues is expected to gain importance. V Henriques and C Hansen [42] compared groups of pigs living at 0 °C: one group with their skin directly exposed to the cold ambient air and the other where animals had a sheepskin garment. After 2 months, bare-skinned pigs had adipose tissues that solidified at a temperature 2.4 °C lower than pigs protected by a coat. This suggests an increase in the degree of desaturation in the FAs of pigs whose skin was in direct contact with the cold environment. Both groups were fed the same food; therefore, this could be either a result of the modification of their FAs or the selective incorporation of certain FAs from the diet to fulfil their thermal needs. Low tissue temperatures, therefore, seem to affect the desaturation of FAs. Conversely, when the skin temperature is maintained close to the warm body temperature, as it is the case of garment-covered pigs and fur-covered mammals, a modification of FAs may not be necessary.

Semi-aquatic mammals, unlike the other two groups, have higher ∆9-DI as they inhabit higher latitudes (Fig. 1b). When hair density was included in the analysis, latitude was still a more important driver of ∆9-DI (Table 2). Conversely, DBI, which included dietary FAs, did not change with latitude, which suggests that this relationship between desaturation of FAs (∆9-DI) and latitude is not due to differences in diet.

The blubber section used for analysis seems to affect at some degree the ∆9-DI, as values of whole blubber cores where usually lower than those of outer layer, for animals living in similar latitudes. Thus, when only the outer layer was used, the correlation between ∆9-DI and latitude for pinnipeds is stronger, with a steeper slope (Fig. 2). However, since the number of data points obtained from whole blubber cores was relatively small (*n* = 6, versus *n* = 25 for all semiaquatic species), this did not alter the correlation found between ∆9-DI and latitude in semiaquatic mammals.

Figure 4 allows us to infer some of the mechanisms behind the increased FA desaturation in colder regions. The FAs 18:1ω9 and 14:0 did not display a relationship with latitude, suggesting that they do not have an important role in the increased FA desaturation of pinnipeds exposed to colder temperatures. The other FAs, however, behaved as expected; the SFAs 16:0 and 18:0 decreased with latitude whereas the MUFAs 14:1ω5, 16:1ω7 and 18:1ω7 increased.

The effect of temperature on FAs has been well documented for organisms at lower trophic levels. The most commonly observed change in FAs following a temperature shift is an alteration in desaturation. In carp fish, for example, the expression of the enzyme that incorporates the first unsaturation bond into saturated FAs, Δ9-desaturase, is induced by cold temperature [43]. Similarly, an increase of FA desaturation when exposed to lower environmental temperatures has been reported for algae and copepods [44–46]. Since mammals exert sensitive control over their desaturases [47], cold-induced mechanisms could be regulating their increase of MUFAs and decrease of SFAs at higher latitudes. Although this broad analysis does not allow us to determine the biochemical paths behind these patterns; we expect that these findings encourage further investigation on the thermoregulatory mechanisms of blubber.

Therefore, why are semi-aquatic mammals the only group that modifies its FAs intrinsically as a response to ambient temperatures? Semi-aquatic mammals are the only ones that move between two environments. Switching from one environment to another implies that animals are exposed to contrasting temperatures and very different heat loss rates. Additionally, they possess both fur and blubber insulation, which is an interesting aspect of mammalian thermoregulation. The presence of dense, waterproof fur is a characteristic of recent entries to the aquatic environment, such as otters, beavers and other rodents [19, 48]. In this study, muskrats, beavers and otters, although living in cold regions, were the species with the lowest degrees of desaturation (Fig. 1b) suggesting that this is a pinniped-only phenomenon. Interestingly, these recent entries to the aquatic medium have the highest hair densities (See Additional file 1: Table S1.). This supports the idea that these mammals rely on dense waterproof fur, rather than the adipose tissue, as an insulator [48]. The efficiency of this coat relies on the air trapped in its hairs [49], which forms a protective warm layer and keeps the skin relatively dry [50]. This thermal barrier, however, can be a disadvantage in swimming performance.

Earlier entries to the aquatic environment, such as pinnipeds, have become much better swimmers, and this has implied the reduction of drag through more streamlined bodies [51], reduction of fur density and thickness [52], and the transition to blubber as an insulator [19]. They possess a wettable fur that is not a good insulator in water, as the air layer trapped in the fur is released due to compression when diving [19]; thus, blubber becomes more important as a thermal barrier.

A thick blubber layer could ensure an effective insulation in water, which is the case of most fully-aquatic mammals, but for a semi-aquatic mammal an extremely thick blubber layer can be problematic. Most semi-aquatic mammals are relatively small compared to cetaceans, and an extremely large blubber layer would impede an agile terrestrial locomotion, especially for those animals living in rookeries with steep slopes, which is common amongst sea lions and fur seals. A trade-off, having a not too thick, but efficient thermal insulator is therefore imperative. A variation in FA composition can be an effective mechanism to maintain a good insulating layer without limiting the manoeuvrability of these animals. Higher FA desaturation in higher latitudes allows the blubber layer near the skin to lower its temperature and therefore reduce heat loss to the surrounding environment due to peripheral vasoconstriction. This feature can be more important in pinnipeds than in any of the other two groups, as they cannot afford a very thick pelage like terrestrial mammals, and neither can they develop extremely thick blubber as fully-aquatic mammals.

Additionally, air temperature variations could explain why semi-aquatic mammals are significantly influenced by latitude and fully-aquatic mammals are not. The water temperature may vary widely, from 30 °C to about − 2 °C, from the tropics to the poles, whereas air temperature in the poles can easily drop to about − 25 °C in winter [53]. Therefore, semi-aquatic mammals are experiencing the most extreme temperature variations and, in absence of an insulating fur coat, the modification of FA desaturation in higher latitudes is a good solution to avoid tissue solidification.

Another interesting aspect of blubber is that it has been reported to display thermal behaviour consistent with phase change materials [54]; thus, using biochemical bonds to store and release heat when lipids melt or solidify at certain temperatures [55, 56]. Many of the FAs found in the blubber are classified as phase change materials [57]; they have melting points between 29° and 38 °C [55] which are within mammalian body temperatures. This suggests that changes in desaturation of FAs might not only be related to pliability but also thermal storage; however, this needs further investigation.

### Dietary influence

The influence of diet in the FA composition of mammals is undeniable, and this is why the use of FAs as trophic markers is gaining scientific attraction [14, 58]. However, there are many factors that can affect the composition of FAs and we show here that the effect of thermal acclimatization is potentially one of them.

To calculate ∆9-DI, we used only those FAs that can be intrinsically synthesised; however, some FAs can be partially endogenous and partially dietary [14]. This is a limitation to this type of analysis. It can be argued that diet still has an influence in the desaturation of FAs. A recent study by SK Abbott, PL Else, TA Atkins and AJ Hulbert [59] shows that rats fed 12 diets showed changes in their MUFA content. In this study, when rats were fed fat that was high in MUFAs the adipose FA composition became increasingly higher in MUFAs, and vice versa. However, there are other examples where diet has had little influence on MUFAs and SFAs. For instance, in humans, PUFAs show a close relationship between dietary intake and adipose tissue whereas SFAs and MUFAs are less closely correlated [60]. Radio-labelling studies showed that grey seals transformed SFAs into MUFAs because the radio-labelled palmitic acid 16:0 they were fed was modified into 16:1 in their blubber [61]. Other authors agree that MUFAs and SFAs are not expected to reflect dietary intake [62]. In this study, there could still be a dietary influence in the FA desaturation of mammals, however this multi-species approach allows us to see that, when examined more broadly, there is an interesting influence of thermoregulation on FAs and that this is a fertile area to investigate further.

## Conclusions

We show that the ability to modify FAs is an important part of mammalian thermal plasticity, especially for mammals living under adverse thermal conditions. We demonstrate that fully-aquatic mammals have increased FA desaturation in their blubber compared to terrestrial mammals, but they do not modify their FAs in different latitudes; they are potentially regulating other parameters of blubber instead. Terrestrial mammals rely mainly on fur as insulator; thus, their adipose tissues have low levels of FA desaturation which does not vary in colder latitudes. Semi-aquatic mammals increase their FA desaturation when living in colder regions, so that they can cool their superficial tissues near freezing temperatures without solidification. The ability to modify their FAs is imperative in semi-aquatic species, as they cannot afford to have extremely thick blubber layers as fully-aquatic mammals since this would compromise their ability to move on land. Additionally, they are exposed to the greatest temperature variations, just like terrestrial mammals, but they lack an insulating fur coat and, therefore, modify their blubber biochemical composition.

## Additional files

Additional file 1: **Table S1.** Data collected across 54 mammalian species. Blubber section indicates whether samples analysed correspond to the whole core of blubber or just the section closer to the skin (outer layer). Data sources are indicated for fatty acid and hair density data. The desaturation index (∆9-DI) and Double bond index (DBI) calculated are also provided. (DOCX 87 kb)

Additional file 2: **Table S2.** Fatty acid composition of 5 mammal species. Samples of the outer blubber layer were collected from stranded animals along the coast of Sydney, Australia. (DOCX 23 kb)

## Data Availability

All data supporting the conclusions of this article are within the paper and its supporting information files.

## Abbreviations

∆9-DI: Desaturation index
AICc: Akaike information criterion (corrected for sample size)
CIs: Confidence intervals
DBI: Double bond index
FA(s): Fatty acid(s)
MUFA(s): Monounsaturated fatty acid(s)
PUFA(s): Polyunsaturated fatty acid(s)
SFA(s): Saturated fatty acid(s)
