# Supplementary material for: From low to high latitudes: changes in fatty acid desaturation in mammalian fat tissue suggest a thermoregulatory role
Source: BMC Evol Biol. 2019 Jul 26;19:155. doi: 10.1186/s12862-019-1473-5 (PMC6659279; doi:10.1186/s12862-019-1473-5)
Supplement: Supplementary file 1 — Table S1. Data collected across 54 mammalian species. Blubber section indicates whether samples analysed correspond to the whole core of blubber or just the section closer to the skin (outer layer). Data sources are indicated for fatty acid and hair density data. The desaturation index (∆9-DI) and Double bond index (DBI) calculated are also provided. (DOCX 87 kb) [file 12862_2019_1473_MOESM1_ESM.docx]

**Additional file 1**

**Table S1:** Data collected across 54 mammalian species. Blubber section indicates whether samples analysed correspond to the whole core of blubber or just the section closer to the skin (outer layer). Data sources are indicated for fatty acid and hair density data. The desaturation index (∆9-DI) and Double bond index (DBI) calculated are also provided.

| **Species name** | **Common name** | **Environment** | **Latitude** | **n (blubber samples)** | **Blubber section** | **∆9-DI** | **DBI** | **Hair density (hairs/mm**^2^) | **Fatty acid data source** | **Hair data source** |
| --- | --- | --- | --- | --- | --- | --- | --- | --- | --- | --- |
| Rangifer tarandus | Reindeer | terrestrial | 78.00 | 23 | whole core | 0.63 | 0.40 | - | CM Pond, CA Mattacks and RH Colby [1] | - |
| Canis lupus | Grey wolf | terrestrial | 65.11 | 2 | outer | 1.02 | 0.62 | - | A Käkëla and H Hyvärinen [2] | - |
| Nyctereutes procyonoides | Racoon dog | terrestrial | 61.38 | 11 | outer | 1.26 | 0.92 | - | A Käkëla and H Hyvärinen [2] | - |
| Sus scrofa | Swine | terrestrial | 38.63 | 72 | outer | 1.29 | 0.82 | - | JK Apple, CV Maxwell, DL Galloway, CR Hamilton and JW Yancey [3] | - |
| Taxidea taxus | American badger | terrestrial | 41.90 | 158 | whole core | 1.30 | 1.10 | - | HJ Harlow and T Varnell [4] | - |
| Ursus arctos | Brown bear | terrestrial | 65.11 | 4 | outer | 1.53 | 0.69 | - | A Käkëla and H Hyvärinen [2] | - |
| Vulpes lagopus | Arctic fox | terrestrial | 71.38 | 4 | whole core | 2.13 | 0.99 | - | TD Shultz and H Ferguson [5] | - |
| Ictidomys tridecemlineatus | 13-lined ground squirrel | terrestrial | 49.50 | 6 | outer | 3.41 | 1.01 | - | ER Price, C Armstrong, CG Guglielmo and JF Staples [6] | - |
| Mephitis_mephitis | Striped skunk | terrestrial | 44.16 | 7 | whole core | 1.20 | 0.86 | - | A-M Mustonen, J Bowman, C Sadowski, LA Nituch, L Bruce, T Halonen, K Puukka, K Rouvinen-Watt, J Aho and P Nieminen [7] | - |
| Mustela vison | American mink | terrestrial | 63.00 | 16 | whole core | 1.57 | 0.76 | - | AM Mustonen, J Asikainen, J Aho and P Nieminen [8] | - |
| Martes zibellina | Sable | terrestrial | 64.00 | 16 | whole core | 1.34 | 0.89 | - | A-M Mustonen and P Nieminen [9] | - |
| Mustela lutreola | European mink | terrestrial | 46.60 | 17 | whole core | 1.38 | 0.96 | - | A-M Koussoroplis, C Lemarchand, A Bec, C Desvilettes, C Amblard, C Fournier, P Berny and G Bourdier [10] | - |
| Mustela putorius | European polecat | terrestrial | 46.60 | 13 | whole core | 0.77 | 1.04 | - | A-M Koussoroplis, C Lemarchand, A Bec, C Desvilettes, C Amblard, C Fournier, P Berny and G Bourdier [10] | - |
| Martes foina | Beech marten | terrestrial | 46.60 | 13 | whole core | 0.39 | 0.78 | - | A-M Koussoroplis, C Lemarchand, A Bec, C Desvilettes, C Amblard, C Fournier, P Berny and G Bourdier [10] | - |
| Felis silvestris | Wildcat | terrestrial | 46.60 | 14 | whole core | 1.29 | 0.98 | - | A-M Koussoroplis, C Lemarchand, A Bec, C Desvilettes, C Amblard, C Fournier, P Berny and G Bourdier [10] | - |
| Castor canadensis | American beaver | semi-aquatic | 65.11 | 4 | whole core | 0.68 | 1.24 | 373.00 | R Käkëla and H Hyvärinen [11] | FE Fish, J Smelstoys, RV Baudinette and PS Reynolds [12] |
| Castor fiber | European beaver | semi-aquatic | 65.11 | 4 | whole core | 0.94 | 1.20 | 324.30 | R Käkëla and H Hyvärinen [11] | W Meyer, J Schmidt, J Kacza, R Busche, HY Naim and R Jacob [13] |
| Ondatra zibethicus | Muskrat | semi-aquatic | 65.11 | 7 | whole core | 0.97 | 1.08 | 396.15 | R Käkëla and H Hyvärinen [11] | FE Fish, J Smelstoys, RV Baudinette and PS Reynolds [12] |
| Lutra lutra | European otter | semi-aquatic | 65.11 | 5 | outer | 1.52 | 1.30 | 699.00 | A Käkëla and H Hyvärinen [2] | W Meyer, J Schmidt, J Kacza, R Busche, HY Naim and R Jacob [13] |
| Phocarctos hookeri | New Zealand sea lion | semi-aquatic | 50.76 | 16 | outer | 1.71 | 1.33 | 21.60 | A Lambert, L Meynier, LC Donaldson, WD Roe and PC Morel [14] | VB Scheffer [15] |
| Cystophora cristata | Hooded seal | semi-aquatic | 54.04 | 32 | whole core | 1.89 | 1.67 | 13.80 | GW Thiemann, SJ Iverson and I Stirling [16] | VB Scheffer [15] |
| Pagophilus groenlandicus | Harp seal | semi-aquatic | 54.04 | 239 | whole core | 1.92 | 1.79 | 16.80 | GW Thiemann, SJ Iverson and I Stirling [16] | VB Scheffer [15] |
| Eumetopias jubatus | Steller sea lion | semi-aquatic | 61.22 | 96 | whole core | 2.13 | 1.46 | 11.60 | CA Beck, LD Rea, SJ Iverson, JM Kennish, KW Pitcher and BS Fadely [17] | [18] |
| Neophoca cinerea | Australian sea lion | semi-aquatic | 33.90 | 1 | outer | 2.29 | 1.77 | - | This study | - |
| Mirounga angustirostris | Northern elephant seal | semi-aquatic | 37.11 | 20 | whole core | 2.32 | 1.15 | 3.70 | DP Noren, SM Budge, SJ Iverson, ME Goebel, DP Costa and TM Williams [19] | VB Scheffer [15] |
| Arctocephalus pusillus | Cape fur seal | semi-aquatic | 31.60 | 2 | outer | 2.35 | 1.90 | - | JP Arnould, MM Nelson, PD Nichols and WH Oosthuizen [20] | - |
| Arctocephalus tropicalis | Subantarctic fur seal | semi-aquatic | 33.90 | 1 | outer | 1.62 | 1.39 | 417.60 | This study | HE Liwanag, A Berta, DP Costa, M Abney and TM Williams [21] |
| Arctocephalus forsteri | New Zealand fur seal | semi-aquatic | 33.90 | 1 | outer | 2.67 | 2.22 | 335.80 | This study | VB Scheffer [15] |
| Halichoerus grypus | Grey seal | semi-aquatic | 60.12 | 3 | outer | 2.80 | 1.94 | 22.20 | R Käkëla, H Hyvärinen and P Vainiotalo [22] | VB Scheffer [15] |
| Erignathus barbatus | Bearded seal | semi-aquatic | 54.04 | 80 | whole core | 2.96 | 1.81 | 28.00 | GW Thiemann, SJ Iverson and I Stirling [16] | VB Scheffer [15] |
| Histriophoca fasciata | Ribbon seal | semi-aquatic | 65.87 | 2 | whole core | 3.53 | 2.45 | 14.50 | GC West, JJ Burns and M Modafferi [23] | VB Scheffer [15] |
| Mirounga leonina | Southern elephant seal | semi-aquatic | 54.83 | 11 | outer | 3.85 | 1.20 | - | NJ Best, CJ Bradshaw, MA Hindell and PD Nichols [24] | - |
| Leptonychotes weddellii | Weddell seal | semi-aquatic | 77.85 | 19 | outer | 3.92 | 1.15 | 13.60 | KE Wheatley, PD Nichols, MA Hindell, RG Harcourt and CJA Bradshaw [25] | VB Scheffer [15] |
| Ursus maritimus | Polar bear | semi-aquatic | 59.51 | 20 | outer | 3.98 | 1.67 | 29.00 | GW Thiemann, SJ Iverson and I Stirling [26] | W Meyer, J Schmidt, J Kacza, R Busche, HY Naim and R Jacob [13] |
| Lobodon carcinophaga | Crabeater seal | semi-aquatic | 64.15 | 20 | outer | 4.39 | 1.75 | 28.20 | A Guerrero and T Rogers [27] | VB Scheffer [15] |
| Hydrurga leptonyx | Leopard seal | semi-aquatic | 64.15 | 24 | outer | 4.51 | 1.48 | 19.80 | AI Guerrero, J Negrete, MEI Márquez, J Mennucci, K Zaman and TL Rogers [28] | VB Scheffer [15] |
| Odobenus rosmarus | Walrus | semi-aquatic | 79.79 | 14 | outer | 4.75 | 1.77 | 1.80 | EG Skoglund, C Lydersen, O Grahl-Nielsen, T Haug and KM Kovacs [29] | VB Scheffer [15] |
| Pusa sibirica | Baikal seal | semi-aquatic | 53.50 | 30 | outer | 5.52 | 1.64 | - | O Grahl-Nielsen, A Halvorsen, N Bodoev, L Averina, L Radnaeva, N Pronin, R Käkelä and E Petrov [30] | - |
| Pusa hispida | Ringed seal | semi-aquatic | 78.91 | 25 | outer | 7.02 | 1.89 | 200.00 | U Strandberg, A Kakela, C Lydersen, KM Kovacs, O Grahl-Nielsen, H Hyvarinen and R Kakela [31] | VB Scheffer [15] |
| Phoca vitulina | Harbour seal | semi-aquatic | 78.33 | 5 | outer | 7.06 | 1.91 | 216.00 | SM Andersen, C Lydersen, O Grahl-Nielsen and KM Kovacs [32] | VB Scheffer [15] |
| Balaenoptera physalus | Fin whale | fully-aquatic | 34.70 | 1 | outer | 2.08 | 1.10 | 0.00 | D Ruchonnet, M Boutoute, C Guinet and P Mayzaud [33] | - |
| Phocoena phocoena | Harbour porpoise | fully-aquatic | 43.21 | 19 | outer | 2.29 | 0.72 | 0.00 | HN Koopman, SJ Iverson and DE Gaskin [34] | - |
| Megaptera novaeangliae | Humpback whale | fully-aquatic | 27.43 | 17 | outer | 2.45 | 1.61 | 0.00 | CA Waugh, PD Nichols, MC Noad and S Bengtson Nash [35] | - |
| Balaena mysticetus | Bowhead whale | fully-aquatic | 70.60 | 18 | outer | 2.62 | 1.54 | 0.00 | SM Budge, AM Springer, SJ Iverson, G Sheffield and C Rosa [36] | - |
| Balaenoptera acutorostrata | Minke whale | fully-aquatic | 65.72 | 22 | outer | 3.17 | 1.39 | 0.00 | E Olsen and O Grahl-Nielsen [37] | - |
| Delphinus delphis | Short-beaked common dolphin | fully-aquatic | 35.27 | 12 | outer | 3.41 | 1.79 | 0.00 | S Quérouil, J Kiszka, AR Cordeiro, I Cascão, L Freitas, A Dinis, F Alves, RS Santos and NM Bandarra [38] | - |
| Monodon monoceros | Narwhal | fully-aquatic | 72.00 | 20 | whole core | 3.74 | 1.04 | 0.00 | GW Thiemann, SJ Iverson and I Stirling [16] | - |
| Caperea marginata | Pygmy right whale | fully-aquatic | 33.90 | 2 | outer | 1.23 | 1.38 | 0.00 | This study | - |
| Physeter macrocephalus | Sperm whale | fully-aquatic | 37.74 | 15 | outer | 3.86 | 0.86 | 0.00 | MJ Walton, MA Silva, SM Magalhães, R Prieto and RS Santos [39] | - |
| Delphinapterus leucas | Beluga whale | fully-aquatic | 79.05 | 7 | whole core | 3.91 | 1.17 | 0.00 | TM Dahl, C Lydersen, KM Kovacs, SF Petersen, J Sargent, I Gjertz and B Gulliksen [40] | - |
| Stenella frontalis | Atlantic spotted dolphin | fully-aquatic | 35.27 | 10 | outer | 3.94 | 1.57 | 0.00 | S Quérouil, J Kiszka, AR Cordeiro, I Cascão, L Freitas, A Dinis, F Alves, RS Santos and NM Bandarra [38] | - |
| Grampus griseus | Risso’s dolphin | fully-aquatic | 33.90 | 1 | outer | 1.58 | 1.00 | 0.00 | This study | - |
| Lagenorhynchus obscurus | Dusky dolphin | fully-aquatic | 17.40 | 5 | outer | 3.99 | 0.97 | 0.00 | O Grahl-Nielsen, JO Krakstad, L Nøttestad and BE Axelsen [41] | - |
| Hyperoodon ampullatus | Northern bottlenose whale | fully-aquatic | 48.15 | 3 | outer | 4.87 | 0.89 | 0.00 | SK Hooker, SJ Iverson, P Ostrom and SC Smith [42] | - |

**References**

1. Pond CM, Mattacks CA, Colby RH: **The anatomy, chemical composition and maximum glycolytic capacity of adipose tissue in wild Svalbard reindeer (Rangifer tarandus platyrhynchus) in winter**. *J Zool (Lond)* 1993, **229**:17-40.

2. Käkëla A, Hyvärinen H: **Site-specific fatty acid composition in adipose tissues of several northern aquatic and terrestrial mammals** *Comp Biochem Physiol A* 1996, **115**(4):501-514.

3. Apple JK, Maxwell CV, Galloway DL, Hamilton CR, Yancey JW: **Interactive effects of dietary fat source and slaughter weight in growing-finishing swine: II. Fatty acid composition of subcutaneous fat**. *J Anim Sci* 2009, **87**(4):1423-1440.

4. Harlow HJ, Varnell T: **Winter changes in fatty acid composition of badger and coyote tissues**. *Comp Biochem Physiol B* 1980, **67A**:211-214.

5. Shultz TD, Ferguson H: **The fatty acid composition of subcutaneous, omental and inguinal adipose tissue in the Arctic fox (Alopex lagopus innuitus)**. *Comp Biochem Physiol B* 1974, **49B**:65-69.

6. Price ER, Armstrong C, Guglielmo CG, Staples JF: **Selective mobilization of saturated fatty acids in isolated adipocytes of hibernating 13-lined ground squirrels Ictidomys tridecemlineatus**. *Physiol Biochem Zool* 2013, **86**(2):205-212.

7. Mustonen A-M, Bowman J, Sadowski C, Nituch LA, Bruce L, Halonen T, Puukka K, Rouvinen-Watt K, Aho J, Nieminen P: **Physiological adaptations to prolonged fasting in the overwintering striped skunk (Mephitis mephitis)**. *Comparative Biochemistry and Physiology Part A: Molecular & Integrative Physiology* 2013, **166**(4):555-563.

8. Mustonen AM, Asikainen J, Aho J, Nieminen P: **Selective seasonal fatty acid accumulation and mobilization in the wild raccoon dog (Nyctereutes procyonoides)**. *Lipids* 2007, **42**(12):1155-1167.

9. Mustonen A-M, Nieminen P: **Fatty acid composition in the central and peripheral adipose tissues of the sable (Martes zibellina)**. *J Therm Biol* 2006, **31**(8):617-625.

10. Koussoroplis A-M, Lemarchand C, Bec A, Desvilettes C, Amblard C, Fournier C, Berny P, Bourdier G: **From aquatic to terrestrial food webs: decrease of the docosahexaenoic acid/linoleic acid ratio**. *Lipids* 2008, **43**(5):461-466.

11. Käkëla R, Hyvärinen H: **Fatty acids in extremity tissues of Finnish beavers (Castor canadensis and Castor fiber) and muskrats (Ondatra zibethicus)**. *Comp Biochem Physiol B* 1996, **113**(1):113-124.

12. Fish FE, Smelstoys J, Baudinette RV, Reynolds PS: **Fur does not fly, it floats: buoyancy of pelage in semi-aquatic mammals**. *Aquat Mamm* 2002, **28**(2):103-112.

13. Meyer W, Schmidt J, Kacza J, Busche R, Naim HY, Jacob R: **Basic structural and functional characteristics of the epidermal barrier in wild mammals living in different habitats and climates**. *Eur J Wildl Res* 2011, **57**(4):873-885.

14. Lambert A, Meynier L, Donaldson LC, Roe WD, Morel PC: **Body regional distribution and stratification of fatty acids in the blubber of New Zealand sea lions: implications for diet predictions**. *J CompPhysiol, B* 2013, **183**(1):145-156.

15. Scheffer VB: **Hair patterns in seals (Pinnipedia)**. *J Morphol* 1964, **115**:291-304.

16. Thiemann GW, Iverson SJ, Stirling I: **Variation in blubber fatty acid composition among marine mammals in the Canadian Arctic**. *Mar Mamm Sci* 2008, **24**(1):91-111.

17. Beck CA, Rea LD, Iverson SJ, Kennish JM, Pitcher KW, Fadely BS: **Blubber fatty acid profiles reveal regional, seasonal, age-class and sex differences in the diet of young Steller sea lions in Alaska**. *Mar Ecol Prog Ser* 2007, **338**:269-280.

18. !!! INVALID CITATION !!! {}.

19. Noren DP, Budge SM, Iverson SJ, Goebel ME, Costa DP, Williams TM: **Characterization of blubber fatty acid signatures in northern elephant seals (Mirounga angustirostris) over the postweaning fast**. *J CompPhysiol, B* 2013, **183**(8):1065-1074.

20. Arnould JP, Nelson MM, Nichols PD, Oosthuizen WH: **Variation in the fatty acid composition of blubber in Cape fur seals (*Arctocephalus pusillus pusillus*) and the implications for dietary interpretation**. *J Comp Physiol B Biochem Syst Environ Physiol* 2005, **175**(4):285-295.

21. Liwanag HE, Berta A, Costa DP, Abney M, Williams TM: **Morphological and thermal properties of mammalian insulation: the evolution of fur for aquatic living**. *Biol J Linn Soc* 2012, **106**(4):926-939.

22. Käkëla R, Hyvärinen H, Vainiotalo P: **Fatty acid composition in liver and blubber of the Saimaa ringed seal (Phoca hispida saimensis) compared with that of the ringed seal (Phoca hispida botnica) and grey seal (Halichoerus grypus) from the baltic**. *Comp Biochem Physiol B* 1993, **105B**(3/4):553-565.

23. West GC, Burns JJ, Modafferi M: **Fatty acid composition of blubber from the four species of Bering Sea phocid seals**. *Can J Zool* 1979, **57**:189-195.

24. Best NJ, Bradshaw CJ, Hindell MA, Nichols PD: **Vertical stratification of fatty acids in the blubber of southern elephant seals (*Mirounga leonina*): implications for diet analysis**. *Comp Biochem Physiol B* 2003, **134**:253-263.

25. Wheatley KE, Nichols PD, Hindell MA, Harcourt RG, Bradshaw CJA: **Temporal variation in the vertical stratification of blubber fatty acids alters diet predictions for lactating Weddell seals**. *J Exp Mar Biol Ecol* 2007, **352**(1):103-113.

26. Thiemann GW, Iverson SJ, Stirling I: **Seasonal, sexual and anatomical variability in the adipose tissue of polar bears (Ursus maritimus)**. *J Zool* 2006, **269**(1):65-76.

27. Guerrero A, Rogers T: **Blubber fatty acid composition and stratification in the crabeater seal, Lobodon carcinophaga**. *J Exp Mar Biol Ecol* 2017, **491**:51-57.

28. Guerrero AI, Negrete J, Márquez MEI, Mennucci J, Zaman K, Rogers TL: **Vertical fatty acid composition in the blubber of leopard seals and the implications for dietary analysis**. *J Exp Mar Biol Ecol* 2016, **478**:54-61.

29. Skoglund EG, Lydersen C, Grahl-Nielsen O, Haug T, Kovacs KM: **Fatty acid composition of the blubber and dermis of adult male Atlantic walruses (Odobenus rosmarus rosmarus) in Svalbard, and their potential prey**. *Mar Biol Res* 2010, **6**(3):239-250.

30. Grahl-Nielsen O, Halvorsen A, Bodoev N, Averina L, Radnaeva L, Pronin N, Käkelä R, Petrov E: **Fatty acid composition of blubber of the Baikal seal Phoca sibirica and its marine relative, the ringed seal P. hispida**. *Mar Ecol Prog Ser* 2005, **305**:261-274.

31. Strandberg U, Kakela A, Lydersen C, Kovacs KM, Grahl-Nielsen O, Hyvarinen H, Kakela R: **Stratification, composition, and function of marine mammal blubber: the ecology of fatty acids in marine mammals**. *Physiol Biochem Zool* 2008, **81**(4):473-485.

32. Andersen SM, Lydersen C, Grahl-Nielsen O, Kovacs KM: **Autumn diet of harbour seals (*Phoca vitulina*) at Prins Karls Forland, Svalbard, assessed via scat and fatty-acid analyses**. *Can J Zool* 2004, **82**(8):1230-1245.

33. Ruchonnet D, Boutoute M, Guinet C, Mayzaud P: **Fatty acid composition of Mediterranean fin whale Balaenoptera physalus blubber with respect to body heterogeneity and trophic interaction**. *Mar Ecol Prog Ser* 2006, **311**(165-174).

34. Koopman HN, Iverson SJ, Gaskin DE: **Stratification and age-related differences in blubber fatty acids of the male harbour porpoise (Phocoena phocoena)** *J Comp Physiol B* 1996, **165**:628-639.

35. Waugh CA, Nichols PD, Noad MC, Bengtson Nash S: **Lipid and fatty acid profiles of migrating Southern Hemisphere humpback whales Megaptera novaeangliae**. *Mar Ecol Prog Ser* 2012, **471**:271-281.

36. Budge SM, Springer AM, Iverson SJ, Sheffield G, Rosa C: **Blubber fatty acid composition of bowhead whales, Balaena mysticetus: Implications for diet assessment and ecosystem monitoring**. *J Exp Mar Biol Ecol* 2008, **359**(1):40-46.

37. Olsen E, Grahl-Nielsen O: **Blubber fatty acids of minke whales: stratification, population identification and relation to diet**. *Mar Biol* 2003, **142**:13-24.

38. Quérouil S, Kiszka J, Cordeiro AR, Cascão I, Freitas L, Dinis A, Alves F, Santos RS, Bandarra NM: **Investigating stock structure and trophic relationships among island-associated dolphins in the oceanic waters of the North Atlantic using fatty acid and stable isotope analyses**. *Mar Biol* 2013, **160**(6):1325-1337.

39. Walton MJ, Silva MA, Magalhães SM, Prieto R, Santos RS: **Fatty acid characterization of lipid fractions from blubber biopsies of sperm whales Physeter macrocephalus located around the Azores**. *J Mar Biol Assoc UK* 2008, **88**(06).

40. Dahl TM, Lydersen C, Kovacs KM, Petersen SF, Sargent J, Gjertz I, Gulliksen B: **Fatty acid composition of the blubber in white whales**. *Polar Biol* 2000, **23**:401-409.

41. Grahl-Nielsen O, Krakstad JO, Nøttestad L, Axelsen BE: **Dusky dolphins Lagenorhynchus obscurus and Cape fur seals Arctocephalus pusillus pusillus: fatty acid composition of their blubber and prey species**. *Afr J Mar Sci* 2010, **32**(2):187-196.

42. Hooker SK, Iverson SJ, Ostrom P, Smith SC: **Diet of northern bottlenose whales inferred from fatty-acid and stable-isotope analyses of biopsy samples**. *Can J Zool* 2001, **79**(8):1442-1454.
