# Supplementary material for: From low to high latitudes: changes in fatty acid desaturation in mammalian fat tissue suggest a thermoregulatory role
Source: BMC Evol Biol. 2019 Jul 26;19:155. doi: 10.1186/s12862-019-1473-5 (PMC6659279; doi:10.1186/s12862-019-1473-5)
Supplement: Supplementary file 2 — Table S2. Fatty acid composition of 5 mammal species. Samples of the outer blubber layer were collected from stranded animals along the coast of Sydney, Australia. (DOCX 23 kb) [file 12862_2019_1473_MOESM2_ESM.docx]

**Additional file 2**

**Table S2:** Fatty acid composition of 5 mammal species. Samples of the outer blubber layer were collected from stranded animals along the coast of Sydney, Australia.

|  | ***Arctocephalus tropicalis***  ***(n = 1)*** | ***Neophoca cinerea***  ***(n = 1)*** | ***Caperea***  ***marginata***  ***(n = 2)*** | | ***Arctocephalus forsteri***  ***(n = 1)*** | ***Grampus griseus***  ***(n = 1)*** |
| --- | --- | --- | --- | --- | --- | --- |
|  | **Mean** | **Mean** | **Mean** | **S.D.** | **Mean** | **Mean** |
| **C14:0** | 3.17 | 3.08 | 5.35 | 1.13 | 3.20 | 4.69 |
| **C16:0** | 9.33 | 14.20 | 9.77 | 2.73 | 13.06 | 11.66 |
| **C18:0** | 3.16 | 1.47 | 2.21 | 0.67 | 3.33 | 3.09 |
| **C14:1** | 0.07 | 0.45 | 0.33 | 0.08 | 0.07 | 1.08 |
| **C16:1ω7** | 2.09 | 11.02 | 4.14 | 1.11 | 3.06 | 11.89 |
| **C18:1ω9** | 20.92 | 27.57 | 15.29 | 3.03 | 22.35 | 23.27 |
| **C18:1ω7** | 2.23 | 3.97 | 1.58 | 0.17 | 2.42 | 2.30 |
| **C20:1ω9** | 0.20 | Tr | 23.91 | 7.54 | Tr | Tr |
| **C22:1ω11** | Tr | 2.34 | 9.08 | 2.22 | Tr | 0.84 |
| **C18:2ω6** | 1.29 | 1.16 | 1.37 | 0.22 | 1.28 | 0.48 |
| **C18:3ω3** | 3.63 | 1.51 | Tr | - | 11.72 | 6.98 |
| **C18:4ω3** | 0.37 | 0.23 | Tr | - | 0.66 | Tr |
| **C20:2ω6** | 0.53 | 0.23 | Tr | - | Tr | 0.53 |
| **C20:4ω6** | 0.27 | 0.14 | Tr | - | Tr | Tr |
| **C20:5ω3** | 0.65 | 2.40 | 2.81 | 0.65 | 2.34 | 1.34 |
| **C22:4ω3** | 0.36 | 0.15 | 3.07 | 0.32 | 3.25 | Tr |
| **C22:5ω3** | 3.27 | 3.79 | 1.69 | 0.30 | 2.92 | 0.53 |
| **C22:6ω3** | 12.57 | 15.27 | 7.78 | 1.67 | 19.03 | 4.68 |
